# U.S. Centers for Disease Control and Prevention launches new chronic kidney disease surveillance system website

**DOI:** 10.1186/1471-2369-14-196

**Published:** 2013-09-14

**Authors:** Tanya Johns, Bernard G Jaar

**Affiliations:** 1 Department of Medicine, Division of Nephrology, Johns Hopkins School of Medicine, 2024 East Monument Street, Suite 2500, Baltimore, MD, 21205, USA; 2 Department of Epidemiology, Johns Hopkins Bloomberg School of Public Health, Baltimore, MD, USA; 3 Welch Center for Prevention, Epidemiology and Clinical Research, Baltimore, MD, USA; 4 Nephrology Center of Maryland, Baltimore, MD, USA

**Keywords:** Chronic kidney disease, Centers for Disease Control and Prevention, Epidemiology, Surveillance, Website

## Abstract

The burden of chronic kidney disease (CKD) is substantial and is associated with poor health outcomes including increase hospitalizations and premature deaths, as well as considerable health care cost. In recognition of this mounting public health problem, the U.S. Centers for Disease Control and Prevention and their collaborators created a national CKD surveillance system. This commentary introduces the national CKD surveillance system and discusses some of its potential uses.

## Introduction

The burden of chronic kidney disease (CKD) is substantial, and is expected to rise. CKD, as defined by a glomerular filtration rate of < 60 ml/min/1.73 m^2^, is prevalent in 14% of the U.S. population, or 44 million individuals [1]. Kidney disease consistently ranks among the top 10 causes of death, and individuals with CKD are also at increased risk of hospitalizations and cardiovascular events [1,2]. In recognition of this mounting public health problem, the U.S. Centers for Disease Control and Prevention (CDC) was congressionally mandated to implement a national CKD surveillance system. Although a federally funded surveillance system for end-stage renal disease has existed for decades (the United States Renal Data System) there was no corresponding monitoring system for CKD. The CDC partnered with well-established researchers in CKD epidemiology from the University of Michigan and the University of California at San Francisco to design and support such a system. The launch of this website (http://www.cdc.gov/ckd) is timely; prior to this, there was no single place to find detailed and comprehensive information on CKD.

The surveillance system focuses on five topics or indicators in CKD in addition to describing its prevalence and incidence: 1) awareness, 2) risk factors, 3) health consequences, 4) process and quality of care, and 5) healthcare system capacity. These indicators are in line with those of *Healthy People 2020*, which are evidence-based, 10-year, national objectives for improving health of all Americans, put forth by the U.S. Department of Health and Human Services [3]. The 10-year goals include reducing the number of new cases of CKD and its complications, disability, death, and economic burden. Furthermore, the surveillance website features a section that is dedicated to tracking the progress towards meeting the *Healthy People 2020* CKD objectives.

## Resource for general public, patients and health providers

The information on the website is available to everyone with the expectation that it will become an essential resource on CKD. The website is designed to be user-friendly and easy-to-navigate for the general public. The information on the site is presented in a number of different formats including maps, figures, charts, summaries, and videos. The CDC, in addition, creates and disseminates a consensus document, the National CKD Fact Sheet, which provides a concise overview on the burden of CKD. Although much of the information may be useful to the general public, a large portion of the material is especially relevant to patients with CKD and their health care providers. For example, there is a section dedicated to describing the burden of established risk factors among patients with CKD. Patients and their providers may also find the health consequences indicator germane, as it addresses some of the more common comorbidities associated with advanced CKD. Risk factor modification and the potential health consequences of chronic kidney disease are two fundamental areas of CKD education, and physicians, particularly primary care providers and nephrologists, should be well versed in these data to have informed discussions with their patients. The information on the website, however, may serve as a reliable adjunct to the patient education that usually takes place in the office or at the bedside. The site also has videos that deal with important topics in kidney disease. For example, one of the featured videos pertains to the use of non-steroidal anti-inflammatory drugs (many of them available over the counter in the United States) — a practice that should be avoided in patients with CKD (with the notable exception of aspirin for cardiovascular disease protection). Unfortunately, a significant number of patients continue to take these medications because they may be unaware of the damaging consequences, or their physicians overlooked their kidney disease. The website also contains information pertinent to special populations, namely children and solid organ transplant recipients with chronic kidney disease. Health care providers and policy makers may find the content on the processes to improve quality and delivery of care particularly significant. In creating this surveillance system, the CDC and their collaborators envisioned that it would lead to measurable improvement in the burden of CKD and quality of CKD care. The surveillance website while inclusive is not the only resource for important CKD information for patients and health care providers. The American Board of Internal Medicine in partnership with Consumer Reports recently invited a number of medical societies including the American Society of Nephrology to identify “Five Things to Question” as part of their Choosing Wisely initiative. These five questions pertain to test and procedures that are commonly done in patients with CKD, which may be unnecessary or potentially harmful and should be preceded by a thorough risk benefit discussion with the patient and their family. For example, the common practice of using peripherally inserted central catheters in patients with CKD could lead to difficulty with appropriate vascular access creation for hemodialysis when necessary, and should largely be discouraged [4].

## Resource for researchers

The surveillance system was developed with help of prominent clinician-scientists, epidemiologists and statisticians involved in kidney disease research. To create the CKD surveillance system, the CDC and their collaborators analyzed a number of data sources, and determined that no one source would be adequate to create a surveillance system for CKD [5]. Information from well-established data sources were utilized for the surveillance system including federal data (Center for Medicaid and Medicare Services, U.S. Census); national registries [U.S. Renal Data System (USRDS), National Health and Nutrition Survey (NHANES), Scientific Registry for Transplant Recipients, American Medical Association]; population-based and prospective cohort studies (Chronic Renal Insufficiency Cohort [6], Chronic Kidney Disease in Children [7], The SEARCH for Diabetes in Youth Study [8], Atherosclerosis Risk in Communities Study [9], Renal Reasons for Geographical and Racial Difference in Stroke [10]); and healthcare system data from the Veterans Health Administration (VHA) system [11]. In this newly created centralized data repository, over 200 interactive charts and figures are available to all researchers. What is particularly unique to this system is the variety of ways in which the burden of CKD is described; information is drawn not only from general population sources such as NHANES and USRDS, but from the VHA, a large health care system. The information is reliable, regularly updated and new measures and indicators are continually being developed. This website aims to be not only the most comprehensive source for background information paramount to CKD research, but also to highlight areas of kidney disease that are lacking in evidence and in need of further research.

There are a number of limitations of the present CKD surveillance system. Although the surveillance system draws from many established data sources, none of these data sources were designed for the specific purpose of CKD surveillance. In using the data from administrative sources and cohort studies, the information is also subjected to the inherent biases, such as lack of representativeness, absence of longitudinal data, missing or incomplete data, and diagnostic inaccuracies. Although inclusion of health system data is unique and enriching, the indicators described within the context of the VHA system may not be representative of all health care systems. The initial intent was to have information from private health care plans, however the creators purport that it was difficult to enlist these sources because of “privacy concerns, time and effort requirements, and cost” [12]. Incorporation of information from the different data sources while improving the representativeness; creates uncertainty regarding the appropriate denominator for the risk estimates, especially with regard to the health system data. Other limitations cited in the executive summary included difficulty getting unpublished data from investigators and having little to no control over some analyses because summary data, rather than raw data, was provided [12].

## Other national surveillance systems

The Kidney Disease Improving Global Outcomes (KDIGO) task force periodically disseminates surveillance data from other countries. These other surveillance systems vary based on the country's health care policies and resources. Some countries, similar to our national surveillance system, rely heavily on established data sources and survey data, while other countries may actively screen for kidney disease and/or create active registries for patients with CKD. However, all these surveillance systems share the same objectives: quantifying the burden of disease; promoting awareness; and implementing policies that will lead to prevention and improved health care to patients with kidney disease [13,14].

## Conclusion

According to the CDC, the purpose of the Chronic Kidney Disease Surveillance System is to “document the burden of CKD and its risk factors in the U.S. population over time and track the progress of our efforts to prevent, detect, and manage CKD” [15]. Such a system is necessary to promote public awareness and advance initiatives that will lead to early detection and prevention, and ultimately reduce the burden of CKD in the U.S. This website will undoubtedly become a paramount resource for patients, physicians, researchers, policy-makers and the public at large*.*

## Abbreviations

CKD: Chronic kidney disease; CDC: Centers for disease control and prevention; USRDS: U.S. renal data system; NHANES: National health and nutrition survey; VHA: Veterans health administration; KDIGO: Kidney disease improving global outcomes.

## Competing interests

The authors declare that they have no competing interests.

## Authors’ contributions

TJ participated in the conception and drafting of the manuscript. BGJ participated in the conception and drafting of the manuscript and revision of important intellectual content. All authors read and approved the final manuscript.

## Pre-publication history

The pre-publication history for this paper can be accessed here:

http://www.biomedcentral.com/1471-2369/14/196/prepub
